# Limb-Salvage Surgery of Soft Tissue Sarcoma with Sciatic Nerve Involvement

**DOI:** 10.1155/2018/6483579

**Published:** 2018-03-06

**Authors:** Hussein Sweiti, Noor Tamimi, Fabian Bormann, Markus Divo, Daniela Schulz-Ertner, Marit Ahrens, Ulrich Ronellenfitsch, Matthias Schwarzbach

**Affiliations:** ^1^Department of Surgery, Clinical Center Frankfurt Höchst, Frankfurt, Germany; ^2^Department of Trauma and Orthopedic Surgery, BG Trauma Center, Frankfurt, Germany; ^3^Institute for Pathology, Clinical Center Frankfurt Höchst, Frankfurt, Germany; ^4^Radiological Institute, Agaplesion Markus Hospital, Frankfurt, Germany; ^5^Department of Hematology and Oncology, University Hospital Frankfurt, Frankfurt, Germany; ^6^Department of Vascular and Endovascular Surgery, Heidelberg University Hospital, Heidelberg, Germany

## Abstract

**Background:**

The surgical resection of soft tissue sarcomas (STS) with sciatic nerve involvement presents a significant surgical and oncological challenge. Current treatment strategies pursue a multimodal approach with the aim of limb preservation. We aim to evaluate the outcomes of limb-sparing surgery of STS in a patient cohort and to propose a classification for STS with sciatic nerve involvement.

**Methods:**

Patients receiving limb-preserving resections for STS with sciatic nerve involvement between 01/2010 and 01/2017 were included. Clinical and oncological data were prospectively collected in a computerized database and retrospectively analyzed. Sciatic nerve involvement in STS was classified preoperatively as follows: type A for nerve encasement; type B for nerve contact; and type C for no nerve involvement.

**Results:**

A total of 364 patients with STS were treated, of which 27 patients had STS with sciatic nerve involvement. Eight patients with type A tumors (29.6%) underwent sciatic nerve resection, and 19 patients with type B tumors (70.4%) received epineural dissections. Disease progression was observed in 8 patients (29.6%) with a local recurrence of 11.1% and distant metastasis in 29.6%. The type of nerve resection significantly influenced leg function but had no impact on disease recurrence or overall survival.

**Conclusion:**

In a cohort of carefully selected patients with STS and sciatic nerve involvement, the extent of sciatic nerve resection had no significant impact on disease recurrence or survival. Precise classification of neural involvement may therefore be useful in selecting the appropriate degree of nerve resection, without compromising oncological outcome or unnecessarily sacrificing leg function.

## 1. Introduction

Soft tissue sarcomas (STS) are a rare and heterogeneous group of mesenchymal tumors, representing only 1% of all adult malignancies [1, 2]. The incidence in Europe has been reported as 4 per 100,000 people per year [3]. These tumors vary in their tendency for aggressive behavior and can occur in all age groups and in a variety of anatomic sites [4]. The lower extremity, however, is the most commonly affected site with approximately 28% of all STS arising there [5]. At least 50 histologic subtypes have been identified, with undifferentiated pleomorphic sarcoma (UPS) and liposarcoma being the most common subtypes [6].

Local disease control is essential in the management of STS, with surgical resection being the only treatment modality capable of achieving complete tumor cell eradication [7]. Achieving negative microscopic margins upon resection of STS has been shown to significantly reduce the risk of local recurrence [8]. The ability to obtain wide margins may however be particularly challenging if the tumor is in close proximity to important neurovascular structures. For STS with vascular involvement, reasonable oncological outcomes have been reported with vessel reconstruction in limb-salvage surgery [9–11]. Nerve reconstruction, on the other hand, does not guarantee preservation of function [12]. Tumor infiltration of the sciatic nerve has previously been an indication for limb amputation [13], but more recent studies have shown limb-sparing surgery with partial or complete sciatic nerve resection to be an excellent alternative [14–17].

The aim of this study is to analyze the oncological and functional outcomes of limb-sparing surgery in STS with sciatic nerve involvement. In addition, we aim to classify the degree of nerve involvement and suggest a suitable therapeutic approach for neural involvement.

## 2. Methods

### 2.1. Study Design, Setting, and Participants

The data of all adult patients with STS (extremities, trunk, and retroperitoneal) undergoing surgical treatment at the Clinical Center Frankfurt Hoechst from January 1st, 2010 until January 31st, 2017 were collected in a computerized database on an ongoing basis and was retrospectively analyzed. Patients with STS of the lower extremity with sciatic nerve involvement who underwent limb-preserving tumor resections were selected from the database and included in this study. All patients consented on the use of their clinical data for research purposes. The study was approved by the ethics committee of the Medical Council of the State of Hesse, Germany.

Involvement of the sciatic nerve was confirmed preoperatively when CT or MRI scans showed no layer of normal tissue between the tumor and the sciatic nerve. Lower limb sarcomas arising from the sciatic nerve or those extending towards the sciatic nerve were included.

### 2.2. Classification of Nerve Involvement

**Figure 1:**
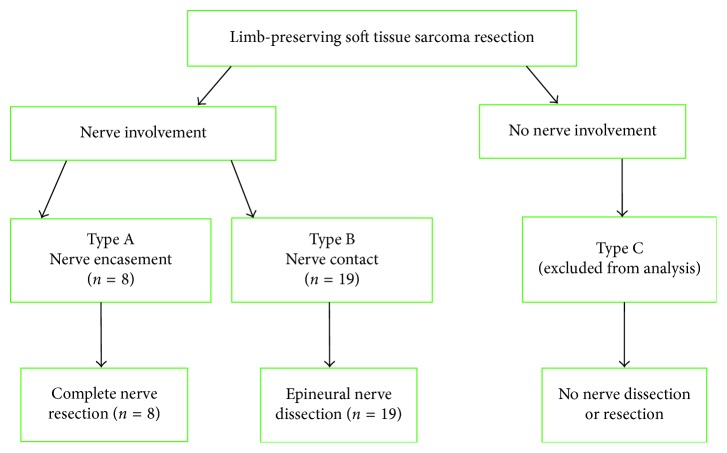
Classification of sciatic nerve involvement and surgical treatment algorithm for lower limb STS.

**Figure 2:**
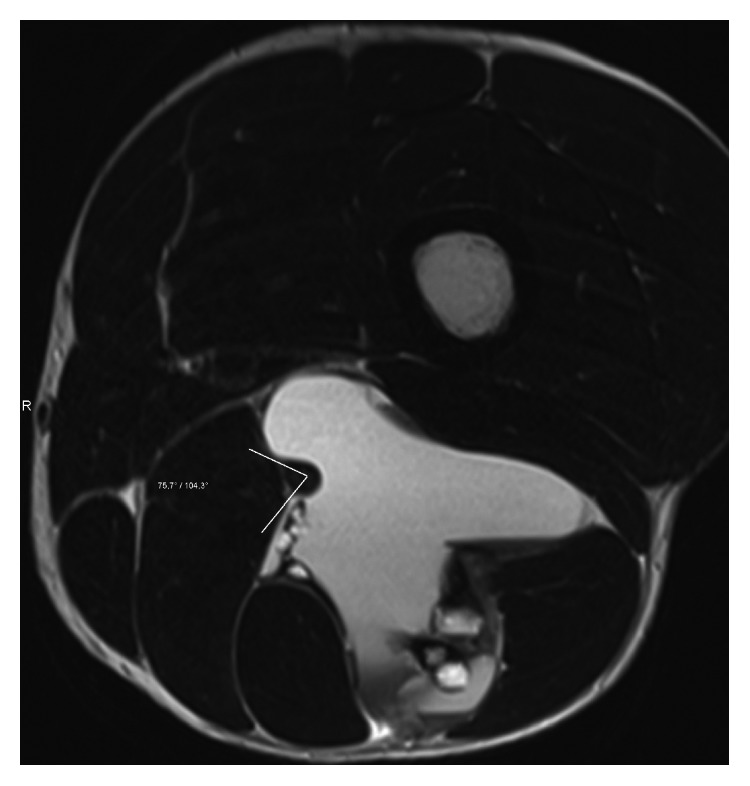
Preoperative MRI scan in a patient with type A sciatic nerve involvement and G3 pleomorphic sarcoma.

**Figure 3:**
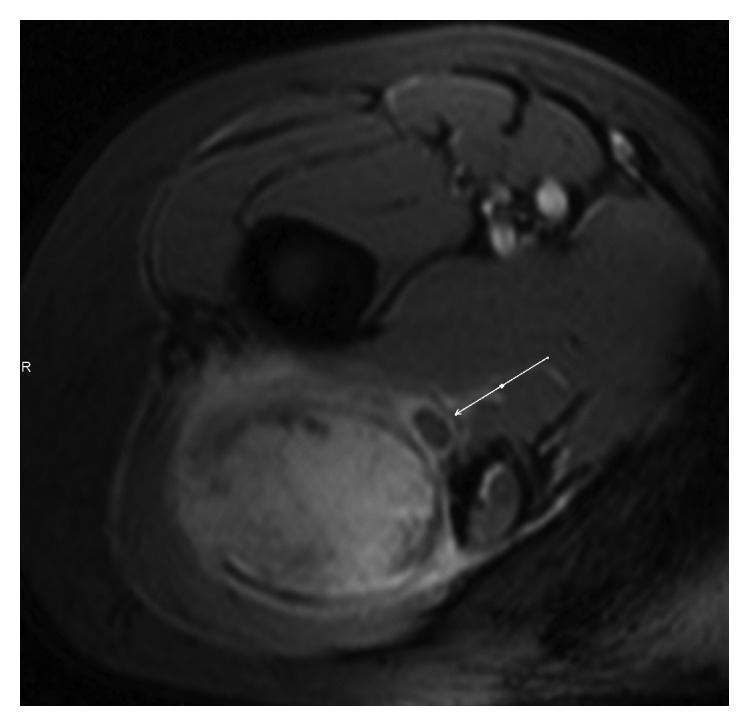
Preoperative MRI scan in a patient with type B sciatic nerve involvement and G2 liposarcoma.

The extent of neural involvement was assessed using high-resolution CT and/or MRI scans. STS with encasement of the nerve were classified as type A. Encasement was defined as ≥180° of nerve contact with the tumor. These tumors were reassessed intraoperatively and underwent en bloc compartmental resection together with the nerve, if the classification was confirmed. STS which revealed direct contact with the nerve (<180°) without encasement or disruption of its continuity were classified as type B and were treated with a compartmental resection of the tumor with epineural dissection. STS without nerve involvement were classified as type C and were resected without nerve dissection or resection (Figure 1). MRI scans from two of our patients illustrating type A and type B sciatic nerve involvement are displayed in Figures 2 and 3, respectively.

Intraoperative reassessment of sciatic nerve involvement was done by visually scrutinizing and palpating the relationship of the nerve to the tumor, if possible. In selected cases, intraoperative ultrasound was employed to visualize the extent of contact of the tumor to the nerve.

### 2.3. General and Perioperative Variables

In addition to basic patient demographic data (age, gender, and affected side), the status of each patient at the time of presentation (primary tumor, local recurrence, and presence of metastasis) was also noted. All therapeutic measures (external radiation therapy, chemotherapy, isolated limb perfusion, or surgical resection) were carried out upon recommendation by a multidisciplinary tumor board. En bloc compartmental resections were carried out in accordance with the surgical standards described by Enneking et al. [18, 19]. Assessment of tumor specimens was carried out by the in-house pathologists and confirmed by the reference pathological department of Heidelberg University Hospital. Specimens were assessed for histological entity, tumor size (maximal diameter), grade, microscopic margins, and nerve infiltration. Tumor grading was based on the criteria of the “Fédération Nationale des Centres de Lutte Contre le Cancer” (FNCLCC), which takes cell differentiation, mitotic activity, and necrosis into consideration. Finally, duration of surgery, surgical and medical complications, reoperations, and the duration of hospital stay were recorded.

### 2.4. Survival, Disease Progression, and Functional Outcome

Following discharge, patients were seen at regular intervals as part of their cancer follow-up care. Patients with intermediate- and high-grade tumors received quarterly clinical exams and MRI studies during the first two postoperative years, every six months during the third year, and on an annual basis afterwards for two more years. Chest CT scans were carried out every six months. Patients with low-grade tumors received clinical exams and MRI studies every six months during the first two postoperative years, and annually for three more years. Chest CT-scans or X-rays were offered on a yearly basis. Information on the functional outcome was recorded by examining the lower limb for function and range of motion (categories: normal, limited, and severely limited). Limited function was defined as a reduced knee flexion of 90°–110° and/or weakness of the intrinsic foot muscles; movement of the foot was possible but reduced. Patients with severely limited function of the leg had a severely reduced knee flexion (<90°), and minimal or no movements of the foot were possible. Patients were also asked about the presence of chronic swelling, paresthesia, or chronic pain as well as their walking range, the use of walking aids, and their satisfaction with limb preservation. Finally, the musculoskeletal tumor society (MSTS) rating score modified by Enneking was calculated in the 20 surviving patients [20]. This scoring system consists of six main categories: pain, limb function, walking aids, walking distance, gait, and emotional acceptance. A score of 0–5 is assigned to each category; higher scores are associated with a greater level of function. The scores out of a total of 30 were then converted to percentages.

### 2.5. Statistical Methods

Statistical analyses were performed with IBM SPSS Statistics 24. Continuous variables were expressed as median and range, and correlations between continuous variables were explored using the Pearson correlation test. The *X*^2^ test and Fischer exact test were used when comparing categorical variables. When comparing categorical variables with continuous variables, the Kolmogorov–Smirnov–Lilliefors test was implemented in determining whether data followed a normal distribution. The independent *t*-Test was used with normally distributed data, and the Wilcoxon–Mann–Whitney *U* test was used with nonnormally distributed data.

The Kaplan–Meier method was used to calculate the survival and disease progression curves, and the log-rank test was used to calculate differences between groups. A *p* value of ≤0.05 was considered significant.

## 3. Results

### 3.1. Participants

A total of 364 patients with STS underwent surgical resection between January 1st, 2010 and January 31st, 2017. The lower extremity was affected in 179 patients (49.2%) and the upper extremity was affected in 19 patients (5.2%). Twenty-seven patients (15.1% of all patients with lower limb STS) had sciatic nerve involvement (type A or B) and were included for further analysis.

### 3.2. Preoperative Characteristics

Descriptive analysis of the 27 included patients revealed a median age of 57 years (interquartile range (IQR): 46–74 years). Six patients (22.2%) presented with a local recurrence while the remaining 21 patients (77.8%) presented with primary tumors. None of the patients presented with primarily metastasized disease. The tumor entity was confirmed in all cases via trucut or incisional biopsy. Based on the proposed neural involvement classification system, 19 patients (70.4%) had STS with direct contact with the sciatic nerve (type B) and 8 patients (29.6%) revealed encasement of the sciatic nerve (type A). Additional general and preoperative characteristics are summarized in Table 1.

### 3.3. Surgical Therapy and Histopathologic Results

All surgical resections of STS were carried out by one experienced surgeon (Matthias Schwarzbach). A macroscopically complete resection without amputation was achieved in all patients. The median operative duration was 5.17 hours (IQR: 3.92–6.54 hours). Eight patients (29.6%) with type A sciatic nerve involvement underwent complete resection of the sciatic nerve, and the remaining 19 patients with type B nerve involvement underwent epineural dissection. The preoperative radiological categorization of type A and type B nerve involvement was confirmed intraoperatively in all 27 cases. Liposarcoma was the most common histopathologic entity (48.1%), with 9 out of 13 liposarcomas diagnosed as low grade (G1). The median tumor size measured by the pathologist following resection was 15 cm (IQR: 8.5–26.5 cm). The negative margin rate in our series was 92.6% with a median margin of 5 mm (IQR: 3–10 mm). Two patients with positive margins (R1) were initially classified as type B. They both received adjuvant radiotherapy and were disease-free at the latest follow-up appointments (22 and 17 months postoperatively).  Table 2 summarizes additional histopathologic findings.

### 3.4. Postoperative Course

Seven patients (25.9%) received adjuvant radiation therapy (60–66 Gy total dose), and one patient (3.7%) received adjuvant radiochemotherapy. Five other patients (18.5%) were subject to adjuvant chemotherapy. A total of 20 patients (74.1%) developed a surgical morbidity, and 6 patients (22.2%) developed a medical complication. Wound-related morbidity, such as necrosis, dehiscence, or infection, was the most common complication affecting 10 patients (37.0%), followed by hematomas or seromas which affected 6 patients (22.2%). In addition, two patients (7.4%) suffered a fracture of the operated extremity following discharge. No hospital mortalities took place, and the median hospital stay was 30 days (IQR: 22–48 days).  Table 3 provides a list of all complications.

### 3.5. Oncological Outcome

**Figure 4:**
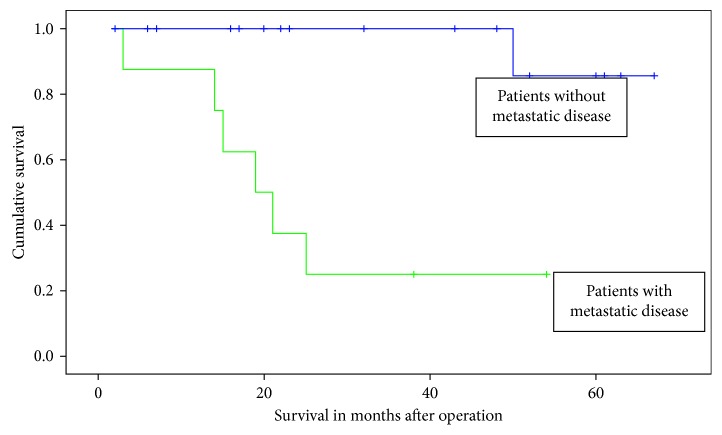
Development of metastatic disease and overall survival (*p* < 0.001).

Patients were followed up for a maximum duration of 5 years postoperatively. The median postoperative follow-up duration was 23 months (IQR: 15.5–50 months). Eight patients (29.6%) were found to have progression of disease (local recurrence or metastasis). All 8 patients had metastatic disease, 3 of which (11.1%) also developed a local recurrence. The most common site of metastasis was the lung, with 5 patients developing pulmonary metastases. A secondary limb amputation was carried out in one patient due to a local recurrence. The overall mortality rate in our series was 25.9% (*n*=7), with a tumor-related mortality rate of 22.2% (*n*=6). A significant association between the development of metastasis and mortality was demonstrated by the Kaplan–Meier survival analysis (*p* < 0.001), as shown in Figure 4.

**Figure 5:**
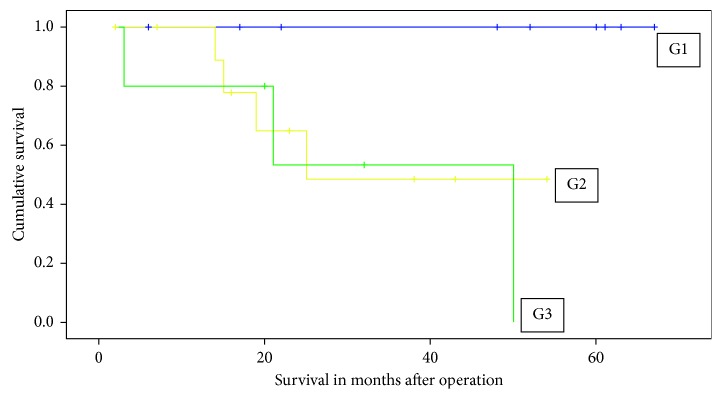
Tumor grade (G1/G2/G3) and overall survival (*p*=0.023).

Various general, perioperative, and histopathologic parameters were investigated for their association with disease progression or mortality. Patient age, initial presentation with recurrent disease, tumor size, tumor histology, type of nerve resection, duration of surgery, and duration of hospital stay were not found to have a statistically significant impact on the development of postoperative complications, disease progression, or survival. Resection margin in millimeters positively correlated with postoperative survival (*p*=0.014). Higher tumor grades (G2 and G3) were significantly associated with the development of distant metastatic disease (*p*=0.010) as well as mortality (*p*=0.020), compared to low grade tumors (G1).  Figure 5 shows the Kaplan–Meier survival curve for different tumor grades (*p*=0.023).

### 3.6. Functional Outcome

The postoperative functional outcome assessment revealed that 50% of surviving patients had an MSTS score of 83% or higher. Five patients (25%) scored between 67% and 80%, and the remaining 5 patients had a score of less than 67%. The main functional outcomes are summarized in Table 4.

Complete sciatic nerve resection was found to be significantly associated with the development of leg edema (*p*=0.017), chronic pain (*p*=0.003), reduced leg function (*p* < 0.001), and lower MSTS scores (*p*=0.001) when compared to epineural nerve dissection. All patients, including those with complications or recurrence of disease, expressed their satisfaction with their decision in opting for limb-sparing surgery as opposed to amputation of the leg.

## 4. Discussion

Our study has shown reasonable oncological and functional outcomes following limb-sparing surgery in a patient cohort with STS and sciatic nerve involvement treated in a specialized center. The frequency of local recurrence (11.1%) and distant metastasis (29.6%) compare well with a large prospective study of 1,041 patients with STS, which reported rates of 17% and 22%, respectively [21]. More recent studies, however, demonstrated local recurrence rates of 10% or less [6, 22–25]. Pisters et al. found that high-grade lesions were a significant prognostic factor in the development of metastatic disease, which was also confirmed in our patient cohort [21].

Liposarcoma and pleomorphic sarcoma were the two most common histopathological entities in our study population, which is analogous to the current literature [6]. The histopathological subtype was not found to be of prognostic significance in our study, which may be due to our small population size. Other studies have shown the histological subtype to be an independent prognostic factor. Resection margins have also been shown to be an independent prognostic factor in local and distant disease control [26–28]. This was confirmed in our study, as the size of the margins was significantly correlated with survival after surgery.

The overall 5-year survival of patients with metastatic STS has been shown to be poor [6]. Our study confirmed the correlation between the development of metastatic disease and mortality, which has been shown in previous studies [21]. Williard et al. reported a tumor-related mortality rate of greater than 50% despite local tumor control, independent of whether patients were treated with limb amputation or limb-sparing surgery, further emphasizing the need to improve systemic disease control [29].

This is a series of large, deep, and in 7 cases recurrent STS with sciatic nerve involvement undergoing compartmental tumor resections as part of a multimodal therapeutic approach. Wound necrosis or dehiscence and the collection of hematomas or seromas were particularly common postoperative complications, occurring in 37.0% and 22.2% of cases, respectively. These factors contributed, in our opinion, to a high reoperation rate of 48.1% as well as a median hospital stay of 30 days.

In the past, some authors recommended hip disarticulation or hindquarter amputation when complete resection of the sciatic nerve was indicated, as a limb without tactile sensations was not considered worth saving from a functional perspective [13, 29–32]. Several authors have, however, reported acceptable functional outcomes after complete resection of the sciatic nerve [11, 15–17, 33], with some studies demonstrating superior function when comparing sciatic nerve resection with amputation of the leg [34, 35]. In our study, all patients expressed their satisfaction with the decision to undergo limb-sparing surgery, despite functional limitations which were particularly apparent in the sciatic nerve resection group. It is important that patients are properly instructed preoperatively regarding adequate foot care of their postoperative insensate feet to minimize skin complications, particularly the development of foot ulcers, which can ultimately lead to a secondary amputation of the limb [16].

The extent of nerve resection was not found to affect the local or distant recurrence probability or have an impact on survival in our study. Similar local recurrence rates were also reported by Clarkson et al. in their cohort of 94 patients when comparing sciatic nerve resection with epineural dissection [17]. Their study also demonstrated superior functional outcomes with patients receiving epineural nerve dissection compared to complete nerve resection. Our study further confirms these findings, as there was a significant association in the development of chronic leg edema, chronic pain, poor leg function, and lower MSTS scores in patients who had undergone a complete nerve resection when compared with nerve dissection. In addition, O'Donnell et al. found that sparing adjacent critical structures did not increase the risk of a local recurrence or reduce survival rates and led to superior functional outcomes in 169 patients with STS and positive margins after tumor resection [36]. We therefore propose that the sciatic nerve is resected only when there is tumor encasement of the nerve (>180°), which is similar to the recommendations made by Clarkson et al. [17].

Our proposed classification system provides a simple and clinically applicable algorithm to facilitate the choice between nerve resection or epineural dissection in patients undergoing limb-sparing surgery due to STS with sciatic nerve involvement. The significance of this classification lies in its potential to encourage a limited epineural dissection in eligible patients (type B) without compromising the oncological outcome or unnecessarily sacrificing the leg function. In addition, this classification may help establish limb-salvage surgery as the procedure of choice in patients requiring complete sciatic nerve resection (type A). The initial assessment of nerve involvement is radiological followed by an intraoperative confirmation. Hence, this classification may be used in the preoperative setting to inform and consent the patient on the expected procedure and its alternatives. It is essential to validate the proposed classification and to critically assess its applicability for different nerves separately, due to variations in their sensorimotor functions and in the degree of compensation following nerve resection.

The present study is one of the largest published series on STS with sciatic nerve involvement to date, as most prior studies were limited to a cohort of less than 20 patients [14–17, 26]. Nevertheless, the small number of patients with this rare constellation of soft tissue sarcoma with sciatic nerve involvement limits the statistical power of our analysis. In addition, the proposed classification does not take significant prognostic parameters, such as grading, into consideration. The tumor grade may influence the extent of surgical resection and could potentially be incorporated into the treatment algorithm. For example, a nerve-sparing surgical resection should be thoroughly considered in a young patient with a well-differentiated liposarcoma and type A sciatic nerve involvement to minimize the loss of function. This is because these tumors rarely metastasize, and the risk of local recurrence may be reduced by incorporating adjuvant or neoadjuvant radiotherapy. In addition, the established classification for vascular involvement in STS by Schwarzbach et al. could also be combined with our proposed classification for nerve involvement, enabling STS with neurovascular involvement to be more accurately classified [9, 37]. Furthermore, the effects of neoadjuvant and adjuvant therapy on both functional and oncological outcomes were not addressed in our study, and no patient-reported functional outcomes were reported in the current series. This data may be used in future studies to compare preoperative and postoperative functions, as it has been suggested that patients with worse function preoperatively have more room to improve postoperatively [15].

## 5. Conclusions

This is the first study to date to classify the extent of sciatic nerve involvement in STS and to suggest a surgical treatment algorithm. In our study, the extent of nerve resection had no significant impact on disease recurrence or overall survival. Hence, precise classification of nerve involvement is useful in selecting the appropriate degree of nerve resection, without compromising oncological outcome or unnecessarily sacrificing leg function. Additional studies are necessary to validate and optimize this classification.

## Figures and Tables

**Table 1:** General and preoperative characteristics.

Characteristic	Number of patients (*N*=27)	%
Gender		
Male	12	44.4
Female	15	55.6
Sides		
Right	16	59.3
Left	11	40.7
Presentation status		
Primary tumor	21	77.8
Local recurrence	6	22.2
Sciatic nerve involvement		
Type A	8	29.6
Type B	19	70.4
Neoadjuvant therapy		
External beam radiation therapy	10	37.0
Chemotherapy	6	22.2
Isolated limb perfusion	5	18.5

**Table 2:** Histopathologic findings.

Characteristic	Number of patients (*N*=27)	%
Histologic entity		
Liposarcoma (all subtypes)	13	48.1
Pleomorphic sarcoma (all subtypes)	11	40.7
Malignant giant cell tumor	1	3.7
Myxofibrosarcoma	1	3.7
Primitive neuroectodermal tumor	1	3.7
Grade		
Low grade (G1)	10	37.0
Intermediate grade (G2)	5	18.5
High grade (G3)	12	44.4
Maximum tumor diameter (cm)		
≥30	5	18.5
20–29	4	14.8
10–19	9	33.3
<10	9	33.3
Margin		
Microscopically negative margins (R0)	25	92.6
Microscopically positive margins (R1)	2	7.4

**Table 3:** Postoperative morbidity.

	Number of patients (*N*=27)	%
Surgical complications		
Wound necrosis/dehiscence	10	37.0
Hematoma/seroma	6	22.2
Fracture (after discharge)	2	7.4
Bleeding	1	3.7
Reoperations (total)	13	48.1
Wound revisions	10	37.0
Hemorrhage control	1	3.7
Reduction and internal fixation	2	7.4
Medical complications		
Pneumonia	2	7.4
Urinary tract infection	2	7.4
Sepsis	1	3.7
Deep venous thrombosis	1	3.7
Hospital mortality	0	0

**Table 4:** Functional outcome.

	Number of patients (*N*=27)	%
Chronic leg edema	15	55.6
Paresthesia	18	66.7
Chronic pain	12	44.4
Walking aids/braces	17	63.0
Leg function/range of motion		
Severely limited/no function	9	33.3
Limited	12	44.4
Normal	6	22.2
Walking distance		
>500 m	15	55.6
100–500 m	9	33.3
<100 m	3	11.1
